# Falls and injuries to Polo players: risk perception, mitigation and risk factors

**DOI:** 10.1186/s40798-014-0002-8

**Published:** 2015-01-20

**Authors:** C M Inness, K L Morgan

**Affiliations:** 1 Department of Musculoskeletal Biology, Institute of Ageing and Chronic Disease, University of Liverpool, Leahurst Campus, Neston, CH64 7TE UK; 2 School of Veterinary Sciences, University of Liverpool, Leahurst Campus, Neston, CH64 7TE UK

## Abstract

Polo, one of the world’s oldest sports, is played in over 80 countries. It is unique in combining the skills of a person with the agility and performance of an animal in a contact sport. There is only one report of the frequency and type of injuries in this population. Here we report risk perception, mitigation and risk factors for injuries and falls in UK polo players. Data were collected retrospectively from a random sample of 112 UK polo players by telephone questionnaire. Injuries (commonly to a shoulder or wrist) requiring a hospital visit were sustained by 17.3% (95% confidence interval [CI] 9.1–25.5) of players. Falls (odds ratio [OR] 6.6, 95% CI 1.4–31.9) and higher self-assessed fitness levels increased the risk (OR 1.7, CI 1.2–2.4). Use of wrist supports (OR 0.2, CI 0.03–0.9) and gym exercise (OR 0.1, CI 0.02–0.9) reduced it. Falls were reported by 58% (CI 47.3–68.8) of players. Women were less at risk than men (OR 0.3, CI 0.1–0.9). Aiming for a better handicap increased the risk (OR 8.4, CI 1.2–57.0). Pre-season rider and horse training were also risk factors. Helmets are compulsory, but players reported that safety certification was not their most important criterion for helmet selection; 49.4% (CI 38.5–60.3) chose appearance. Attendance of a doctor at polo games was not considered important by 65.4% (CI 55.1–75.8) of players; attendance of paramedics and ambulances was volunteered as being of greater consequence. The findings of this study suggest that the protective effect of wrist supports needs testing, helmet manufacturers should incorporate both style and safety into their designs, and paramedics and ambulances should attend polo games.

## Key points

- This is the first study of the perception of risk, mitigation and risk factors for falls and injuries in polo players.
- Injuries requiring hospital attendance were sustained by 17.3% of players; higher self-assessed fitness levels increased the risk; use of wrist supports and gym exercise reduced it.
- Falls were reported by 58% of players; aiming for a better handicap increased the risk; pre-season rider and horse training reduced it.

## Background

Polo, one of the world’s oldest sports, is unique in merging the skill of a person with the agility and performance of an animal to create a single participant in a contact sport. In this respect, it differs from all other sports. Preventing injuries in these modern-day centaurs offers medics, vets and dentists an unrivalled opportunity to collaborate, but surprisingly there has only been one previous publication on injuries in this population [1]. We have recently reported the prevalence and risk factors for injuries in polo ponies [2]. Here we present the risk and type of injury, risk factors and perception of risk, training and fitness in riders in the same population.

Polo is played in over 80 countries. Two teams of four mounted players hit a 9 cm white plastic ball, at speeds of up to 96 miles per hour, using the wide face of a 48- to 54-inch-long polo mallet with a bamboo shaft and a hardwood head. A goal is scored when the ball passes, at any height, between two posts at each end of the pitch. The game is divided into 7.5 min time periods called ‘chukkas’. There can be between four and eight chukkas per game. The winning team is the one that has scored the most goals when adjusted for player handicap. Handicaps range from −2 to 10 and are summed to give a combined team handicap. The lower handicapped team is awarded goals at the start of the game, depending on the handicap difference. Polo is played in arenas and outdoors. In the UK, outdoor polo is played on grass pitches from the start of April until the end of September. Membership of the governing body, the Hurlingham Polo Association (HPA), is obligatory. Currently, almost 3,000 full playing members and over 13,000 horses are registered.

Polo is considered a high-risk sport, but in the only previous report, a small prospective study of elite players in Argentina, the incidence of injury was 7.8/1,000 playing hours [1]. This is relatively low when compared with soccer (17–29/1,000 h) [3-5] and rugby (53.8/1,000 h) [6]; however, 64% of these injuries were considered serious. Arm and head injuries were most common and were frequently associated with falls [1]. The only other information on injuries in polo players is clinical reports of unusual cases, such as ulna stress fracture [7] and popliteus muscle rupture [8].

Polo has been advocated as a sport for all ages [9]. The starting point for a ‘one health’ [10] approach to injury prevention is to determine the frequencies and risk factors for injuries in riders and horses [2] in the same population. Riders’ opinions on causes of injury, their perceptions, and mitigation of risk and risk factors are reported here.

## Methods

### Study design

Telephone interviews were used to collect data on injuries, falls, pre-season training and perceptions of risk to polo owner-players and horses, using a retrospective cohort design. The study unit was the player. Potential participants were contacted by mail and were informed of the study aims and that taking part was voluntary, opinions and data were anonymous and opting out was a prerogative. Telephone interviews were conducted between 26 April and 4 May 2010. The option to opt out was repeated at the beginning of each interview. Any person who was unavailable during this period or was non-contactable after five attempts was considered a non-responder. One person conducted all interviews. The University of Liverpool Veterinary Ethics Committee approved the study.

### Study population

The target population was HPA-member, UK-resident, horse-owning polo players, aged over 18 years, with an address and telephone number in the members’ database. These criteria were met by 1,181 players. A random sample of 112 was selected without replacement. Players known personally to the interviewer (CMI) were substituted with another randomly selected member. The sample size was calculated to allow estimation of the prevalence of injuries, falls and opinions with 95% confidence and 10% precision, assuming the population prevalence to be 50% and a predicted response rate of 80% [11]. This was estimated to be 89 players. This sample size allowed 95% confidence that one opinion, variable or injury would be detected if its prevalence was 3%.

### Questionnaire design

The questionnaire design is detailed elsewhere [2]. Briefly, it had two sections—one related to players, the other to the horses that they owned. It contained closed and open questions. Player–owners were asked about falls and acute injuries sustained in the previous season. Injuries were defined as physical insults sustained during a polo game that required a hospital visit. Respondents were asked about their height, weight and handicap; the range and intensity of their winter exercise; the importance of pre-season training on a 1 to 4 scale (with 1 being the lowest); their self-reported fitness levels on a 1 to 10 scale (with 10 being the highest); their perception of the risk of injury in the first, middle and final thirds of the season; their use of protective apparel; and their opinion on the importance of a doctor attending games, on a 1 to 4 scale. The questionnaire was pre-tested before use and modified accordingly, but there was no external validation.

### Data processing and analysis

Data were entered into a Microsoft Access database. Frequency distributions of injuries were described, and exact binomial 95% confidence intervals (CIs) were calculated. Falls and any rider injury were used as binary variables, and the association with different exposure variables was examined. Univariable analyses were carried out using Epi-Info 2007 [12]. Continuous variables were compared using analysis of variance, except where Bartlett’s test indicated variance heterogeneity, when Kruskal–Wallis tests were used. Categorical variables were analysed using *χ*^2^ and Fisher’s exact tests.

All variables associated with either falls or injuries with *p* values of <0.1 in univariable analyses were offered to multivariable logistic regression models developed manually using forward stepwise procedures in Stata [13]. Variables were added in the order of their *p* value, smallest first. Any with *p* values of <0.05 were retained in the model. Variables with a value approaching 0.05 were retained where the change in the Akaike information criterion (AIC) supported it. Model quality was assessed by the sensitivity, specificity, accuracy and area under the receiver–operating characteristic (ROC) curve and the goodness of fit evaluated by the Hosmer–Lemeshow statistic.

## Results

### Response rate

The useable response rate was 73% (81/112). Eighty-four polo players responded, but two no longer kept polo ponies and one had kept them in work over winter.

### Participants

The participants’ mean age was 41.7 years (standard deviation [SD] 10.7), the mean body mass index (BMI) was 24.8 (SD 1.9) and the median handicap was 0 (interquartile range [IQR] 0–1). Each participant owned between 2 and 110 horses, with a median of seven (IQR 5.0–10.0). Eighty percent (CI 71.6–88.9) of respondents were men. Sixty-three percent (CI 52.5–73.5) were responsible for day-to-day horse management, and the remainder employed a groom.

### Falls

Fifty-eight percent (CI 47.3–68.8) of players reported falling in the previous season. The median frequency was once (IQR 0–2), but 2.5% (CI 0.0–5.9) of players fell 10 or more times.

### Injuries to riders

Fourteen riders (17.3%, CI 9.1–25.5) reported injuries. Wrist and shoulder injuries were most common. The injuries were a broken collar bone (2), shoulder injury, wrist injury (3), damage to tendons in a hand and wrist, dislocated elbow, torn groin ligament, broken rib, ‘damaged cartilage’, damage to cruciate ligaments, damaged right leg with torn calf muscle and haematoma, pelvic injury x-rayed but not broken, hamstring injury, and arm and fingers hit by a mallet. Three patients reported more than one injury. Players were hit in the mouth by mallets (9.9%, CI 3.4–16.4) and balls (3.7%, CI 0.0–7.8). None required dental treatment. All but 1.2% (CI 0.0–3.6) of players reported bruising after playing, and 56.8% (CI 46–67.6) were bruised regularly.

### Pre-season exercise and fitness levels

Just over half of the respondents (54.3%, CI 43.5–65.2) rode during winter, 37% (CI 26.5–47.6) played winter polo and 86.4% (CI 79.0–93.9) reported gym exercise, hockey, running, swimming, yoga, squash, weight training, Pilates, cycling and walking either singly or in combinations. The frequencies of these different types of exercise are shown in Table 1.

**Table 1:** **Frequencies of winter exercise and use of protective apparel reported by players**

	**Number**	**Percentage**	**95% CI**
Winter exercise			
Gym exercise	33	40.7	30.0–51.4
Running	18	22.2	13.2–31.3
Swimming	14	17.3	9.1–25.5
Yoga	9	11.1	4.3–18.0
Squash	8	9.9	3.4–16.4
Weight training	7	8.6	2.5–14.8
Pilates	6	7.4	1.7–13.1
Cycling	5	6.2	0.9–11.4
Walking	3	3.7	0.0–7.8
Use of protective apparel			
Knee pads	80	98.8	96.4–100
Goggles	40	49.4	38.5–60.3
Wrist support	28	34.6	24.2–44.9
Face guard	17	21.0	12.1–29.9
Elbow pads	8	9.9	3.4–16.4
Gum shield	5	6.2	0.9–11.4
Back support	3	3.7	0.0–7.8

*CI* confidence interval.

Over half of the players (58.0%, CI 47.3–68.8) considered their exercise intensity to be less than the recommended UK adult level of ‘moderate exercise for 30 min or more five times per week’. Their median self-assessed fitness to play polo was 5 (IQR 4–7) with a range of 2–10.

When asked for their opinion on the importance of pre-season training, 58% (CI 47.3–68.8) scored it a very important 4; 35.8% (CI 26.5–47.6) scored it 3; 4.9% (CI 0.2–9.7) scored it 2 and 1.2% scored it 1.

### Use of protective apparel

The use of protective apparel is shown in Table 1. Wearing a helmet is compulsory in the UK, and all respondents reported wearing one. The next most common item was knee pads, followed by goggles, wrist supports, face guards, elbow pads, gum shields and back supports.

### Perception of risk

Although it is compulsory for helmets to carry a safety standard mark, only 29.6% (CI 19.7–39.6) of riders considered it the most important aspect of helmet purchase, 49.4% (CI 38.5–60.3) chose appearance and 7.4% (CI 1.7–13.1) chose price.

Perception of risk changed with the stage of the season. Forty-three percent (CI 32.4–54.0) of respondents considered it highest in the first third of the season, compared with 1.2% (CI 0.0–3.6) in the last third. Thirty-seven percent (CI 26.5–47.6) considered the risk constant throughout the season.

The presence of doctors at games was considered of low or very low importance by the majority of participants (65.4%, CI 55.1–75.8), but a significant minority (23.5%, CI 14.2–32.7) considered it highly important. Many participants volunteered the opinion that the attendance of paramedics and an ambulance was of far greater consequence.

### Risk factors for injury

Risk factors for serious rider injury are shown in Table 2. Rider age, sex, height, weight, BMI and experience did not influence the risk of injury. In univariable analysis, risk factors were a fall, higher self-scored fitness, participation in gym exercise and yoga, use of wrist supports and the number of days from the start of the year to the first chukka.

**Table 2:** **Risk factors for serious rider injury**

**Risk factor**	**Odds ratio**	**95% CI**	***p*** **value**
Fall	6.57	1.35–31.91	0.02
Rider self score of fitness	1.68	1.18–2.40	0.004
Rider exercise: gym	0.14	0.23–0.88	0.035
Rider use of wrist supports	0.17	0.30–0.93	0.04

*CI* confidence interval.

After adjustment for confounding, falls were associated with an increased risk of injury (odds ratios [OR] 6.6, CI 1.4–31.9), as was higher self-scored fitness (OR 1.7, CI 1.2–2.4). In contrast, gym exercise reduced the likelihood of injury (OR 0.14, CI 0.03–0.9), as did use of wrist supports (OR 0.17, CI 0.02–0.9). Use of goggles and face protectors had no effect.

The model had sensitivity of 50%, specificity of 92.5%, accuracy of 85.2% and an area under the ROC curve of 0.87. The Hosmer–Lemeshow goodness-of-fit statistic was 4.2 (*p* = 0.84).

### Risk factors for falls

Risk factors for falls during polo games are shown in Table 3. In univariable analysis, risk factors for falls were the sex of the rider, yoga and squash as out-of-season exercise, aiming for a lower handicap, cold water hosing of the horse’s legs after exercise and the duration of pre-season horse training.

**Table 3:** **Risk factors for falls during polo games**

**Risk factor**	**Odds ratio**	**95% CI**	***p*** **value**
Rider sex: female	0.27	0.08–0.94	0.04
Rider aim to increase handicap	8.36	1.23–56.97	0.03
Rider exercise: yoga	20.73	1.64–262.59	0.02
Rider exercise: squash	NA	Perfect predictor	
Horse: pre-season exercise (per day)	0.93	0.88–0.98	0.012
Horse: cold water hosing	6.10	1.47–25.41	0.013

*CI* confidence interval, *NA* not applicable.

After adjustment for confounding, the odds of women falling was a third of that of men (OR 0.3, CI 0.1–0.9). Players who aimed to improve their end-of-season handicap had eight times the risk (OR 8.4, CI 1.2–57.0) of those who did not aim to improve their handicap. All eight riders who reported playing squash experienced at least one fall, and those who reported doing yoga had 20 times the risk (OR 20.7, CI 1.6–262.6) of those who did not report doing yoga.

Players exercising their horses for longer prior to the first chukka (51.1 versus 43.5 days) were less likely to fall; the OR was 0.93 for every day increase. Riders who cold-water hosed their horses’ legs after exercise had six times the risk of falling (OR 6.1, CI 1.5–25.4).

This model had sensitivity of 80.9%, specificity of 73.5%, accuracy of 77.8% and an area under the ROC curve of 0.87. The Hosmer–Lemeshow goodness-of-fit statistic was 7.92 (*p* = 0.44). When squash was excluded from the model, the AIC was 89.8, compared with 82.7 when squash was included; the latter was selected.

## Discussion

This is the first description of the frequencies and risk factors for injuries and falls in UK polo players. It also records their perceptions of risk and its mitigation.

Polo is recognised as a rough contact sport [1]. Almost all of the players in this study reported bruising after playing. Injuries requiring a hospital visit were less common. The cumulative risk was 17.3% (CI 9.1–25.5), which equates to 519 hospital visits per year in the total population. Shoulder and wrist injuries were most common. This finding is consistent with the only previous report of polo injures [1].

Use of wrist supports was associated with a decreased risk of injury (OR 0.17, CI 0.03–0.93). This applied to all injuries rather than just those involving the wrist. Interpreting this as a proxy measure of risk-averse or safety-conscious riders is not supported by the lack of association between wrist supports and falls. Wrist supports may reduce the effect of forces on the wrist caused by swinging the mallet. This hypothesis is biologically plausible and warrants an intervention study.

Rider fitness was associated with positive and negative risks; perception of self fitness was associated with an increased risk of injury, whereas actual fitness, as measured by gym exercise, was associated with a decreased risk.

Rider falls were common, and they were associated with injury (OR 6.6, CI 1.4–31.9). Fifty-eight percent of players reported falling at least once during the season. Risk factors were the sex of the rider, intention to improve handicap, pre-season pony exercise and days from the start of the year.

Females were half as likely to fall as males (OR 0.3, CI 0.1–0.9). This may be because women are better riders, have better balance, or are more cautious and risk averse during games. It could not be explained by physical attributes, such as height and weight, or riding experience, as falls were not associated with these variables.

Players aiming to improve their handicap were 8.4 (CI 1.2–56.9) times as likely to fall as those who considered that their handicap would remain the same. This may reflect more competitive players pushing themselves and exceeding their limits in order to improve. It was not associated with rider age.

The association between rider falls and the number of days of pre-season pony exercise was a surprise. Riders exercising horses for a mean of 51.1 days (SD 12.2) were at lower risk (OR 0.9, CI 0.9–0.98) of falling than those exercising for 43.5 days (SD 10.9). This suggests that horse preparation is important in preventing rider falls and may reflect improved fitness, reduced excitability or better control. Alternatively, it may be a proxy measure for another variable, e.g. management. It is also possible that this effect was associated with the improved ground conditions later in the calendar year, rather than the time interval itself. When the number of days from the start of the year to the first chukka was examined, it was associated with a decrease in falls at a level close to statistical significance (*p* = 0.052). It is possible that two effects operate here: pre-season pony training and improved ground conditions. The effect of pre-season pony exercise needs further investigation.

Attitude to risk was assessed from priorities in helmet selection, the frequency of wearing protective apparel, perceived periods of highest risk and the perceived importance of attendant medical staff.

Helmets are compulsory, but only 29.6% (CI 19.7–39.6) of riders chose safety as the most important criterion for selection. Almost half of them considered appearance most important. This highlights the need for style and safety considerations in helmet design [14].

The ranking of additional protective apparel was knee pads, goggles, wrist supports and face guards. A small proportion of players used elbow pads, gum shields and back supports.

Players differed in their perception of the stage of the season and the risk of injury. The first third of the season was considered high risk by 43.2% (CI 32.4–54.0), whereas a similar proportion (37.0%, CI 26.5–47.6) considered the risk constant throughout the season. A prospective study would add quantitative data to these perceptions.

Rider opinions on the value of having a doctor present were polarized, with 65.5% scoring it low priority and 23.5% scoring it high priority. The only other study of polo injuries recommended the presence of a trauma-trained doctor at all matches [1]. Although it was not on the questionnaire, the importance of paramedic and ambulance attendance was voiced by a number of riders.

This is the first population-based study of falls and injuries to polo players in the UK. It is small because of the absence of any recognizable funding stream for research on polo, but it followed STROBE (Strengthening the Reporting of Observational Studies in Epidemiology) guidelines to maximize accuracy and generalizability. The usable response rate was lower than anticipated but at 73% was still acceptable. A retrospective cohort design was used, and to maximize recall, the study focused on memorable events, injuries requiring a hospital visit and falls in the previous season. In the UK, this would involve a visit to an Accident and Emergency Department, and this was used as a proxy measure for the injury being both acute and serious. We did not ask for precise details of the injuries, their severity or the injury mechanism, as our focus in this initial study was simply to estimate injury frequency and type. The study was carried out at the beginning of the season because we were also interested in out-of-season training of riders and horses. The results are player reports. Although there was no external validation, the risk of rider injury was similar to that reported previously [1]. Not all respondents were responsible for the day-to-day management of their horses. Thirty-seven percent (CI 26.5–47.6) employed a groom. This may have resulted in inaccuracies but should not have introduced systematic bias.

These UK players differed from those in the only previous report, a prospective study of 34 Argentinean riders [1]. Handicaps ranged from −1 to 7 compared with 7–10 [1], suggesting that the UK players were recreational rather than high performance. At 41.7, the mean age was surprisingly high. This may reflect the inclusion criterion of owning at least one horse. Younger players may have been excluded because their mounts were hired or registered under a parent’s name. Horse-owning players were selected because we were also interested in the injuries to horses [2]. It was considered unlikely that players using hired or loaned horses would be able to supply this information. Selecting this population may have biased the overall estimate of injury by excluding younger players.

Polo is interesting from a number of scientific perspectives. The unique relationship between rider and horse in this ancient, unpredictable contact sport offers medics, vets and dentists the opportunity to collaborate in the field of sports injury prevention.

### Ethical approval

This study was approved by the University of Liverpool Veterinary Ethics Committee and was performed in accordance with the ethical standards laid down in the 1964 Declaration of Helsinki and its later amendments. All participants gave their informed consent prior to their inclusion in the study.

## References

[1] Costa-Paz M, Aponte-Tinao L, Muscolo D (1999). Injuries to polo riders: a prospective evaluation. Br J Sports Med.

[2] Innes CM. Polo pony injuries: player–owner reported risk, perception, mitigation and risk factors. Equine Vet J. Epub 2014 Jul 17. doi:10.1111/evj.12298. 10.1111/evj.12298 24819255

[3] Engstrom B, Johansson C, Tornkvist H (1991). Soccer injuries among elite female players. Am J Sports Med.

[4] Rahnama N, Reilly T, Lees A (2002). Injury risk associated with playing actions during competitive soccer. Br J Sports Med.

[5] Walden M, Hagglund M, Orchard J, Kristenson K, Ekstrand J (2013). Regional differences in injury incidence in European professional football. Scand J Med Sci Sports.

[6] Fuller C, Brooks J, Cancea R, Hall J, Kemp S (2007). Contact events in rugby union and their propensity to cause injury. Br J Sports Med.

[7] Clark R, Sizer P, Slauterbeck J (2002). Stress fracture of the ulna in a male competitive polo player. Am J Sports Med.

[8] Winge S, Phadke P (1996). Isolated popliteus muscle rupture in polo players. Knee Surg Sports Traumatol Arthrosc.

[9] Vail J (2009). Polo for all ages. J Psychosoc Nurs.

[10] Morris A (2009). One medicine: combining veterinary and medical expertise. Vet Rec.

[11] Brogan D, Denniston M, Liff J, Flagg E, Coates R, Brinton L (2001). Comparison of telephone sampling and area sampling: response rates and within-household coverage. Am J Epidemiol.

[12] Dean A, Arner T, Sunki G (2011). Epi-Info™, a database and statistics program for public health professionals.

[13] StataCorp (2009). Stata statistical software: release 11.

[14] Czarnecki N, Yoganandan N, Sances A, Pintar F, Reinartz J, Battocletti J (1986). An evaluation and collective review of helmet standards. J Clin Eng.

